# Pulmonary Function in Pulmonary Sarcoidosis

**DOI:** 10.3390/jcm12216701

**Published:** 2023-10-24

**Authors:** Qian Yao, Qiuliang Ji, Ying Zhou

**Affiliations:** 1 Department of Clinical Research Unit, Shanghai Pulmonary Hospital, Tongji University School of Medicine, Shanghai 200443, China; 2 Department of Pulmonary and Critical Care Medicine, Shanghai Pulmonary Hospital, Tongji University School of Medicine, Shanghai 200433, China

**Keywords:** sarcoidosis, pulmonary function, diagnosis

## Abstract

The pulmonary function test (PFT) has been widely used in sarcoidosis. It may vary due to the severity, extent, and the presence of complications of the disease. Although the PFT of most sarcoidosis patients is normal, there are still 10–30% of cases who may experience a decrease in the PFT, with a progressive involvement of lungs. Restrictive ventilatory impairment due to parenchymal involvement has been commonly reported, and an obstructive pattern can also be present related to airway involvement. The PFT may influence treatment decisions. A diffusing capacity for carbon monoxide (DLCO) < 60% as well as a forced vital capacity (FVC) < 70% portends clinically significant pulmonary sarcoidosis pathology and warrants treatment. During follow-up, a 5% decline in FVC from baseline or a 10% decline in DLCO has been considered significant and reflects the disease progression. FVC has been recommended as the favored objective endpoint for monitoring the response to therapy, and an improvement in predicted FVC percentage of more than 5% is considered effective.

## 1. Introduction

Sarcoidosis is a systemic granulomatous disorder of unknown etiology that may affect almost every body organ. The most commonly involved organs remain the lung and mediastinal lymph nodes. The diagnosis of it depends on a compatible clinical and imaging feature, histologic evidence of non-necrotizing epithelial granuloma, and exclusion of alternative causes of granulomatous diseases [1]. Clinicians have been involved with the disease activity and severity of sarcoidosis through clinical symptoms, radiological imaging, the pulmonary function test (PFT), and blood tests. The PFT plays an important role in the initial workup, diagnosis, and treatment monitoring, as well as follow-up of the disease. It is sensitive for detecting pulmonary parenchymal changes, airway obstruction, and pulmonary hypertension in sarcoidosis. In this review, we evaluated the evidence published in this area to summarize the role of the PFT in initial and follow-up evaluation, the correlation of the PFT with radiological findings, disease severity, and the scoring system for sarcoidosis, as well as the influence of the PFT in treatment. 

## 2. Methods

The PubMed database was searched for the period from January 2012 to December 2022 using the terms: “Sarcoidosis[title]”. The inclusion criteria included: (1) the article was about pulmonary sarcoidosis rather than other organs’ sarcoidosis; and (2) the article contained the content of the FPT. The exclusion criteria included: (1) the article was written in languages other than English; (2) case reports, study designs, comments or letters; (3) animal or laboratory studies; and (4) the full text was unavailable. According to the inclusion and exclusion criteria, there were 45 articles selected by investigators. An additional 39 articles were found by searching the reference lists of previously selected articles. 

## 3. Results

### 3.1. PFT in the Initial and Follow-up Evaluation of Sarcoidosis

The PFT, as well as radiographic and laboratory biomarkers tests, is useful for initial assessment in sarcoidosis diagnosis [2]. The American Thoracic Society (ATS), European Respiratory Society (ERS), and World Association for Sarcoidosis and Other Granulomatous Disorders (WASOG) guidelines recommend a series of preliminary examinations, including the PFT, for all patients with sarcoidosis [2]. Among the PFT parameters, forced vital capacity (FVC), forced expiratory volume in the first second (FEV1), and diffusing capacity for carbon monoxide (DLCO) are necessary for assessing lung involvement in clinical practice. FVC is the most important parameter for monitoring diseases and is often used as the primary endpoint in clinical trials of sarcoidosis [3]. The FEV1/FVC ratio may be effective in identifying most sarcoidosis patients with airway obstruction [4]. DLCO appears to be more sensitive in detecting pulmonary fibrosis than FVC, and it can be used to indicate pulmonary hypertension [5,6]. However, DLCO is not as effective as FVC as a treatment indicator or a primary research endpoint due to its high variability. 

The PFT in sarcoidosis patients may vary due to the severity, extent, and the presence of complications of the disease. Most sarcoidosis patients have a normal PFT, whereas 10–30% of cases may experience a decrease in the PFT, with progressive involvement of the lungs and progress to chronic disease [7]. An impaired PFT at the onset of sarcoidosis has been implicated in poor prognosis in the long term. A value of FVC < 80% was associated with persistence of activity, while a vital capacity (VC) of <1.5 L implied a high risk of mortality [8,9]. Viskum et al. found that patients with a FEV1 lower than or equal to 50% predicted had 4.2-fold increase in mortality rate compared with those with a FEV1 exceeding 80% predicted [10]. Any PFT pattern can be seen in patients with sarcoidosis, such as a restrictive ventilatory defect (RVD), an obstructive ventilatory defect (OVD), reduced DLCO, or mixed ventilatory defects. The most prevalent pattern of PFT abnormality was an RVD due to parenchymal involvement, which occurred in about 45% of the patients [11]. An obstructive pattern can also be present and may be related to airway involvement caused by external compression of mediastinal disease, granulomatous tissue, or peri-bronchial fibrosis [12,13]. In the stage IV sarcoidosis group, spirometry and DLCO are almost always abnormal. An RVD was observed in approximately two-thirds of cases, while an OVD was observed in one-third. Mixed defects were present in 20% of patients, and a decrease in DLCO was observed in 90% of patients [14]. 

The clinical course of sarcoidosis is usually evaluated and tracked with objective clinical outcome measures, including radiographic findings and a PFT [15]. The PFT is the gold standard indicator in evaluating pulmonary parenchymal progression and treatment response. Despite the lack of consensus on follow-up times, it is recommended to conduct an assessment at least every 3 to 6 months in the first 2 years, and yearly for the next 3 to 5 years; thereafter, no further follow-up is required unless recurrence or new symptoms occur [2]. The PFT reflects the effectiveness of treatment. Out of various pulmonary function parameters, FVC is routinely used to assess the response to therapy due to its high reproducibility [16]. Changes in FVC during follow-up are important, and a significant reduction in FVC is an indication for therapy. An absolute change of 5% in FVC is considered significant, and has been proposed as one of the criteria for exacerbation of sarcoidosis [3,17]. Meanwhile, an absolute improvement of FVC > 5% is considered as a positive response to treatment [18]. The FEV1 is related to the severity of airway obstruction. The FEV1 and FEV1/FVC decrease can be seen in sarcoidosis patients with bronchial distortion, peripheral lymph node compression of the airway, or endobronchial involvement [12,19]. After successful therapy, improvement in the FEV1 may be seen in these cases. 

### 3.2. Correlation between PFT and Chest Imaging in Sarcoidosis

X-ray is the most common radiologic technique for assessing pulmonary involvement in sarcoidosis. There are differences in PFT parameters of sarcoidosis patients at different imaging stages [20]. The PFT is impaired in approximately 20% of sarcoidosis patients with stage I, but in 40–80% of stage II to IV patients, with parenchymal involvement [21]. Stage I disease was associated with mild PFT abnormalities, which were better than those of stages II and III, whereas patients at stage IV had the worst pulmonary function and 75% of them died from respiratory complications including pulmonary hypertension and chronic respiratory failure [14,22,23,24]. However, the initial Scadding stage was not well correlated with changes in pulmonary function or subsequent clinical recovery, apart from stages 0 and 4, which were related to great and poor prognosis, respectively [25,26]. A study from the United Kingdom conducted serial chest X-rays (CXR) and simultaneous PFT tests in 354 patients with sarcoidosis, and found that the PFT data of 50% patients were inconsistent with the chest X-ray data, which suggested that disease extent on chest radiography was more appropriate for routine monitoring of sarcoidosis than the X-ray Scadding stage [27].

**Figure 1:**
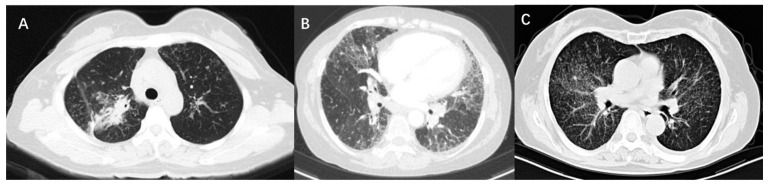
CT images of three patients with sarcoidosis. (**A**) Shows consolidation on CT scan and the PFT is characterized by restrictive ventilation dysfunction, with an FVC of 67.9% predicted and FEV1 of 61% predicted. (**B**) Shows fibrosis and bronchial distortion on CT scan and the PFT is characterized by restrictive ventilation dysfunction and decreased diffusion function, with an FVC of 44.4% predicted, FEV1 of 52.0% predicted, and DLCO of 50.9% predicted. (**C**) Shows multiple micronodules on CT scan and the PFT is characterized by mixed ventilation dysfunction, with an FVC of 71.3% predicted, FEV1 of 62.3% predicted, FEV1/FVC of 69.46%, and normal DLCO.

High-resolution computed tomography (HRCT) is an accurate modality to identify mediastinal lymphadenopathy and subtle pulmonary parenchymal changes. In clinical practice, it is widely used for the initial evaluation of sarcoidosis and monitoring of disease progression. Compared with CXR stages, HRCT findings of sarcoidosis have a better correlation with the severity of PFT changes [23,28]. PFT parameters were negatively correlated with CT scores of consolidation pattern and ground-glass opacities. There were obvious correlations between lung consolidation imaging scores and FVC, FEV1, and FEV1/FVC, while the ground-glass opacity scores were significantly related to DLCO [22,29]. As for micronodules, whether lung function is affected depends on the amount of micronodules and the extent of lung involvement. It was suggested that the higher the number of micronodules is, the lower the spirometric values are [29]. If micronodule patterns occur in a very limited lung area, PFT parameters will not be affected [22]. The main CT features of pulmonary fibrosis included honeycombing patterns, diffuse linear patterns, and bronchial distortion. Honeycombing patterns are usually associated with restrictive dysfunction and decreased DLCO. Patients with bronchial distortion often experience a lower FEV1 and FEV1/FVC. Linear patterns generally only cause slight functional damage [28]. Figure 1 shows the CT images of three sarcoidosis patients with different pulmonary dysfunctions. 

Airflow limitation was observed in patients with thickening of bronchovascular bundles (BVBs), air trapping, and reticular shadow [12,30,31]. Handa et al. conducted a prospective, observational study, and found that 8.8% (20/228) of the sarcoidosis subjects had airflow limitation, and chest radiographic stage IV, higher age, smoking, and thickened BVB were independently associated with a lower FEV1/FVC [12]. Hansell et al. evaluated CT scans of 45 patients with semi-quantitative scoring for five CT patterns. The range of the reticular pattern is closely correlated with airflow obstruction severity. The larger the extent of the reticular pattern is, the lower the values of FEV1 and FEV1/FVC are [30]. Another study considered that air trapping patterns on HRCT were related to PFT parameters in patients with pulmonary sarcoidosis. In that study, 20/21 patients had air trapping patterns. The extent of air trapping patterns was negatively related to the percentage of predicted residual volume (RV) to total lung capacity (TLC), and the percentage of predicted maximal mid-expiratory flow rate between 25 and 75% to VC [31]. 

Fluorodeoxyglucose positron emission tomography (FDG-PET) is a useful tool to evaluate inflammatory activity. The metabolic activity of pulmonary parenchyma displayed by FDG-PET was associated with PFT parameters and may represent an impaired pulmonary function [32,33,34]. In a study, pulmonary PET positive patients had lower DLCO and FVC compared with pulmonary PET negative patients, and PET positivity was observed in all patients with decreased lung function parameters of DLCO < 45% or FVC < 50% [32]. Patients with active pulmonary PET and impaired lung function may have a positive response to treatment. Keijsers et al. found that patients with parenchymal metabolic activity imaged by PET had an obvious increase in lung function of VC, FEV1 and DLCO after treatment, while PET negative subjects showed no change in PFT parameters [35]. Meanwhile, the maximum standardized uptake value (SUVmax) of PET at baseline can predict clinical improvement in pulmonary function after treatment. A prospective open-label trial was performed to evaluate infliximab efficacy in sarcoidosis patients whose symptoms were refractory to conventional treatment in a clinical setting. After 26-week therapy, infliximab significantly improved FVC (6.6% predicted) in refractory sarcoidosis patients with positive 18F-FDG PET [36]. A similar conclusion was reached in another study, which correlated 18F-FDG PET during infliximab treatment with standard sarcoidosis activity parameters, and concluded that the reduction in the SUVmax in pulmonary parenchyma was related to the improvement of VC [37]. 

### 3.3. Evaluation of Severe Sarcoidosis

Severe sarcoidosis may lead to significant disability or reduced life expectancy. Pulmonary fibrosis, impaired lung function, extensive disease on HRCT, and pulmonary hypertension are related to poor clinical outcomes in sarcoidosis patients [38]. Most sarcoidosis patients go into remission spontaneously or after treatment, but up to 20% of patients will develop pulmonary fibrosis [39]. Cough, dyspnea after exercise, and hypoxemia are common clinical symptoms. The most common abnormalities of the PFT in sarcoidosis patients with pulmonary fibrosis are an RVD and a decrease in diffusion capacity, while airflow obstruction caused by central airway fibrosis can also be seen [28,39]. An extension of pulmonary fibrosis greater than 20% on CT is associated with poor survival [5,40]. Therefore, the best strategy is to identify those patients who will develop pulmonary fibrosis early and prevent them from developing advanced diseases by focusing on the progression of respiratory symptoms and changes in PFT parameters, mainly the deterioration of FVC and DLCO. 

Sarcoidosis-associated pulmonary hypertension (SAPH), a late complication of sarcoidosis, is most common in stage IV or advanced disease, but can also occur in the condition of relatively normal lung function and preserved parenchymal architecture. Approximately 5–6% of pulmonary sarcoidosis patients will develop SAPH, and it is a predictor of a worse outcome with a five-year survival rate of 55% [41,42]. Patients with SAPH usually have an advanced chest radiographic stage and decreased pulmonary function. DLCO is useful in evaluating suspected pulmonary hypertension. Pulmonary hypertension (PH) should be suspected when DLCO is reduced or the symptoms of unexplained dyspnea are persistent, especially when DLCO decreases disproportionately compared with pulmonary volumes, with a FVC/DLCO ratio > 1.6 [43,44,45,46]. In a 6-minute walk test (6MWT), DLCO levels < 60% and oxygen saturation (SpO2) < 90% were independently related to the presence of PH, and the level of potential PH increased sevenfold [15,47]. A screening echocardiogram is recommended in these situations. In an international registry study of SAPH patients, the factors related to reduced transplant-free survival have been analyzed with long-term follow-up. Reduced DLCO < 35% predicted and a 6-minute walk distance < 300 m at registration have been considered as powerful predictors of decreased survival [48]. 

### 3.4. Scoring System for Sarcoidosis

Several comprehensive scoring methods have been developed to assess the severity of pulmonary sarcoidosis and guide treatment. Wells and his colleagues designed a composite physiological index (CPI), which is a weighted index of pulmonary function variables, and is related to the extent of disease on HRCT in idiopathic pulmonary fibrosis (IPF), and they confirmed that a CPI can predict mortality more than any single pulmonary function variable in IPF [49]. The calculation formula for the CPI is as follows: 91.0 − (0.65 × percent predicted DLCO) − (0.53 × percent predicted FVC) + (0.34 × percent predicted FEV1). Combining CPI (< or >40) and HRCT variables, including the main pulmonary artery diameter to ascending aorta diameter ratio (MPAD/AAD) (< or >1) and the extent of fibrosis (< or >20%), Walsh et al. established a clinical staging system for rapid risk prediction of sarcoidosis, which was considered more accurate than any single variable alone [5]. 

The sarcoidosis treatment score (STS) system has been developed to assess treatment efficacy based on multiple factors of pulmonary sarcoidosis. This STS system integrates six variables, including 5% of absolute FVC change, 10% of absolute DLCO change, HRCT variations, King’s sarcoidosis questionnaire, the fatigue assessment scale, and changes of daily glucocorticoid dose [50,51]. Each positive change is scored 1 point, while negative change is scored −1 point, with a total score of −6 to 6 points. A score of ≥3/6 is considered as Response (R), a score of 2/6 points is considered as Partial Response (PR), while a score of ≤1/6 is considered Non-Response (NR). The components of the STS have a good correlation, with 5% of absolute change in predicted FVC and 10% of absolute change in predicted DLCO [51]. Recently, this STS system has been successfully validated as a primary study endpoint in a multicenter clinical trial [52]. 

Pulmonary function parameters can also be applied to clinical phenotype identification. A cluster analysis has been studied to phenotype sarcoidosis subjects with slight or severe manifestation [53]. Six phenotypes of sarcoidosis were produced by this cluster analysis. Clusters 1, 2, and 3 had a normal PFT, and cluster 1 was in Scadding stages 2/3, cluster 2 in stages 0/1, and cluster 3 between stages 0/1 and 2/3. Compared with clusters 1, 2, and 3, patients in clusters 4, 5, and 6 had at least one reduced PFT parameter, and needed more therapy. Poorer lung function performances in severe phenotype clusters 4, 5, and 6 were presented as an obstructive type with Scadding stages 2/3, restrictive type with stages 2/3, and mixed types with stage 4, respectively. It is a clinically useful way for clinicians to identify patients with more slight or more severe conditions.

### 3.5. The 6-Minute Walk Distance (6MWD) and Cardiopulmonary Exercise Testing (CPET)

The 6MWD is a simple indicator for measuring pulmonary and cardiac status of patients with sarcoidosis, and it is useful for evaluating exercise tolerance and oxygen demand. It has been confirmed that the 6MWD decreases in some patients with pulmonary sarcoidosis. The 6MWD is most commonly used in the initial assessment, and it is also often used as one of the secondary endpoint indicators in clinical trials of sarcoidosis and as a predictive indicator of the patient survival rate. 

In a prospective study with 142 patients, Baughman and his colleagues assessed the role of the 6MWD in impairment and prognosis of disease, and found that 73 (51%) patients had a 6MWD < 400 m and 32 (22%) patients had a 6MWD < 300 m. Meanwhile, they found that the active ingredients of the St George’s Respiratory Questionnaire (SGRQ), FVC, as well as minimum oxygen saturation were independent predictors of 6MWD [54]. In another observational study, Pescaru et al. found that patients with sarcoidosis had reduced exercise capacity assessed by the 6MWD compared with healthy controls, and observed there was obvious associations between the 6MWD and PFT parameters, including FEV1, FVC and DLCO [55]. 

The 6MWT has been usually tested as one of the secondary endpoints in sarcoidosis therapy clinical research. The 6MWD improved from 227 m to 240 m after six months’ treatment in some patients with SAPH [56]. In another retrospective study of patients with SAPH, the 6MWD increased by 59 m (*p* = 0.032) after specific therapies [57]. This was also confirmed in patients with pulmonary sarcoidosis. A randomized and double-blind study found the 6MWD significantly improved in an infliximab treatment group compared with the placebo group (+8 versus −34.1) [58]. However, the 6MWD is considered to be influenced by several factors involving other lung diseases, cardiac diseases, or muscle involvement, which makes it difficult to identify the reasons for a decrease in the 6MWD and monitor therapy response in some cases [59]. 

The CPET has been considered as a useful tool for assessing exercise tolerance, and it offers added value in detecting impaired PFT in pulmonary sarcoidosis patients [60]. A comparative study found CT findings were correlated with a significant amount of variance in CPET parameters [61]. Compared with the 6MWT, the CPET shows no obvious difference in parameters of HR and SPO2. It could be used as a suitable method in pulmonary sarcoidosis patients, except those with advanced stages [62].

### 3.6. Decision Making and Evaluation of Treatment

The PFT may influence treatment decisions. Most sarcoidosis patients do not require treatment when they have no obvious symptoms, normal PFT parameters, or a high possibility of remission. A cohort study demonstrated that oral glucocorticoids, disease-modifying antirheumatic agents (DMARDs), or biologic agents were required only in 104/311 of pulmonary sarcoidosis cases [63]. However, an obvious and rapid decline in PFT parameters indicated active granulomatous inflammation and progressive disease that might lead to worse outcomes if left untreated. The statements of ATS/ERS/WASOG suggest that systemic treatment in time is necessary for sarcoidosis cases with obvious symptoms, progressive decline of lung function, and persistent pulmonary infiltrate [2]. FVC is the greatest PFT parameter for treatment decisions for pulmonary sarcoidosis, while DLCO provides useful information when the value is significantly lower in percent predicted. At diagnosis, FVC < 70% and DLCO < 60% portend clinically significant pulmonary sarcoidosis pathology, which warrants treatment [15,64]. During follow-up, development of symptoms or an objective loss of pulmonary function reflects the progression of the disease. Treatments should be considered when FVC significantly decreases by 5% from baseline or DLCO decreases by 10% [17]. 

Of the PFT parameters, FVC is recommended as a favored objective indicator for evaluating the response to therapy [3]. The commonly approved treatment goal is to improve the predicted FVC percentage by more than 5% [3,18,51]. Table 1 shows the information about FVC improvement after treatment in several clinical studies. 

Oral glucocorticoids are the first-line therapeutic approach for sarcoidosis patients [2,71]. The therapeutic dose was usually initiated with 0.5–1 mg/kg of prednisone, tapered slowly by 10 mg per 4 weeks, to 5–10 mg/day maintenance. Generally, treatment could be stopped after 6 to 12 months if patients’ symptoms and PFT parameters improved, while the period of treatment needed to be extended to 24 months in refractory disease [72]. Randomized controlled trials have showed that glucocorticoid treatment could improve FVC and DLCO in stage II and III sarcoidosis patients compared with those on placebo. However, no benefit was observed in the glucocorticoid treatment of asymptomatic stage I sarcoidosis subjects, and there was evidence to suggest therapy with glucocorticoids could result in a higher possibility of relapse [67,73]. In a meta-analysis of clinical trials of corticosteroid treatment in sarcoidosis patients, a significant difference in FVC of 4.2% and DLCO of 5.7% of predicted values was observed compared with untreated patients [74]. A multi-center, prospective and observational study in the Netherlands demonstrated that the improvement in FVC occurred within one month after prednisone therapy initiation in newly treated sarcoidosis patients, with an improvement in predicted FVC of 11.8% [69]. Similar results were obtained in another study. An increase of 7.4% predicted FVC at 3 months and 9.6% predicted at 12 months were seen after prednisone therapy, and the improvement in FVC mainly occurred in the first 1–3 months of treatment [70]. 

Methotrexate (MTX) is a preferred second-line medication for sarcoidosis patients [75]. According to the ATS/ERS/WASOG statements, the addition of MTX was suggested to improve pulmonary function or quality of life if glucocorticoids were ineffective or led to unacceptable side effects [71]. Various studies have found that MTX is associated with improved lung function and may help with steroid sparing [76,77,78,79,80]. Lower and Baughman performed a non-randomized clinical study on patients with chronic symptomatic sarcoidosis to determine the efficacy and safety of methotrexate. The authors found that 35 out of 50 patients (70%) showed an improvement in FVC of greater than 10% after at least 2 years of MTX treatment [79]. Azathioprine (AZA) is used as an alternative second-line medication in the treatment of sarcoidosis, but there is no randomized controlled study assessing its efficacy and safety in sarcoidosis. An international retrospective study has been conducted to evaluate MTX and AZA as a second-line treatment. The results showed that both agents had similar effects, with an obvious improvement in the PFT in 70% of patients and steroid-sparing capacity, while patients in the AZA treatment group had a higher infection rate [77]. Mycophenolate mofetil (MMF) may be beneficial for some patients with sarcoidosis, but research results are controversial. In a retrospective study from Switzerland, Brill found that MMF treatment could decrease the maintenance dose of corticosteroids to under 10 mg/day, and improved the lung function, with a median FVC change of +8.5% [81]. However, another retrospective study from the United States demonstrated there was no statistically significant change in PFT measurements before and after MMF treatment [82]. 

Biologic agents are considered as the third-line treatment for patients with refractory diseases or those who cannot tolerate glucocorticoids and other immunosuppressants [64,71]. Infliximab is a humanized monoclonal antibody that neutralizes TNF-α, and has the most robust data for the treatment of sarcoidosis. In a randomized controlled study including 138 cases with chronic pulmonary sarcoidosis, intravenous infusions of infliximab were compared with placebo, and it was found that the predicted FVC in the infliximab treatment group increased by 2.5% at 24 weeks, while the placebo group did not improve [68]. Similar results were noted in a prospective study. Patients with refractory FDG-PET-positive pulmonary sarcoidosis had a 6.6% increase in predicted FVC after being given infliximab treatment at 26 weeks [36]. 

## 4. Conclusions

The PFT is a widely available and useful method for evaluating and managing sarcoidosis. The review of available data suggested the baseline PFT could provide an estimate of disease severity, and a series of PFTs provide valuable information for monitoring disease progression as well as assessing the response to treatment. DLCO < 60% as well as FVC < 70% portends clinically significant pulmonary sarcoidosis pathology, which warrants treatment. During follow-up, a 5% decline in FVC from baseline or a 10% decline in DLCO is considered significant and reflects the disease progression. The improvement in the predicted FVC percentage by more than 5% is considered effective to therapy. In the future, the STS as a key endpoint should be widely used and further optimized in a sarcoidosis clinical study.

## Figures and Tables

**Table 1:** Forced vital capacity (FVC) as a measure of clinical outcomes in sarcoidosis.

First Author (Ref.)	Year	Treatment	Duration	Improvement in FVC % Pred	Improvement in FVC (L)
Anne Pietinalho [65]	1999	oral prednisolone + inhaled budesonide	3 months + 15 months	11.4%	0.15 L
RM du Bois [66]	1999	inhaled fluticasone propionate	6 months	Not Reported	0.08 L
Anne Pietinalho [67]	2002	oral prednisolone + inhaled budesonide	3 months + 15 months	Not Reported	0.33 L
Robert P Baughman [68]	2006	intravenous infusions of infliximab	24 weeks	2.5%	Not Reported
Adriane D M Vorselaars [36]	2015	intravenous infusions of infliximab	26 weeks	6.6%	Not Reported
Caroline E. Broos [69]	2018	oral prednisone	1 months	11.8%	Not Reported
Caroline E. Broos [70]	2018	oral prednisone	12 months	9.6%	Not Reported

## Data Availability

Not applicable.
